# Clinicopathological characteristics of rectal multiple neuroendocrine neoplasms and literature review

**DOI:** 10.1186/s12893-023-02050-2

**Published:** 2023-06-02

**Authors:** Xiuli Zheng, Mingli Wu, Shengmian Li, Limian Er, Huiyan Deng, Shuo Guo, Zhihuan Liu

**Affiliations:** 1 Department of Endoscopy, Fourth Hospital of Hebei Medical University, No. 12 Jiankang Road, Chang’an District, Shijiazhuang, 050000 Hebei China; 2 Department of Gastroenterology, Fourth Hospital of Hebei Medical University, No.12 Jiankang Road, Chang’an District, Shijiazhuang, 050000 Hebei China; 3 Department of Pathology, Fourth Hospital of Hebei Medical University, No. 12 Jiankang Road, Chang’an District, Shijiazhuang, 050000 Hebei China

**Keywords:** Rectal neuroendocrine neoplasm, Rectal multiple neuroendocrine neoplasms, Lymph node metastasis

## Abstract

**Background:**

There are only a few epidemiological reports available for reference. The clinicopathological features are not clear, so there is no consensus on treating rectal multiple neuroendocrine neoplasms. This study aims to summarize the clinicopathological characteristics and preliminarily discuss the clinical diagnosis and treatment of rectal multiple neuroendocrine neoplasms*.*

**Methods:**

This study retrospectively analyzed rectal neuroendocrine neoplasm patients diagnosed and treated at the Fourth Hospital of Hebei Medical University from February 2007 to May 2021. The clinicopathological characteristics of rectal multiple neuroendocrine neoplasms were summarized and analyzed in combination with 14 studies on rectal multiple neuroendocrine neoplasms.

**Results:**

The incidence of RM-NENs accounted for 3.8% of all R-NENs in this study. The number of tumors varied to some extent, the size of tumors was basically no more than 10 mm, and there were more G1 grade tumors. In the analysis of 46 cases with known lymph node metastasis, the difference in lymph node metastasis rate between the number of tumors < 8 and ≥ 8 was statistically significant (*p* = 0.002).

**Conclusions:**

The incidence of rectal multiple neuroendocrine neoplasms accounted for 3.8% of all rectal neuroendocrine neoplasms. For rectal multiple neuroendocrine neoplasms, the lymph node metastasis rate was higher when the number of tumors was ≥ 8. The influence of the number of tumors on lymph node metastasis should be considered in the selection of treatment.

## Introduction

In recent years, the incidence of rectal neuroendocrine neoplasms (R-NENs) has shown an obvious increasing trend, which is probably related to the progress of clinical medicine, the improvement of health awareness and the popularity of colonoscopy [1]. Meanwhile, the disease has gradually gained people’s attention. It has been reported that R-NENs are more common in Asian populations [2] and have become the second-most common neuroendocrine tumors in China [3].

R-NENs usually present as submucosal lesions and yellow mucosa [4, 5]. The treatment and prognosis of rectal single neuroendocrine neoplasm (RS-NEN) are relatively well known [6]. The prognosis of patients with small RS-NEN without lymph node metastasis or distant metastasis is favorable. Although the tumors in the majority of R-NEN cases are single focal tumors, there have been reports of multiple focal tumors [7–11]. According to the existing statistics, the incidence of rectal multiple neuroendocrine neoplasms (RM-NENs) is 2% ~ 5.7% [8, 12], indicating the rarity of the disease. Unlike RS-NEN, there are no standard guidelines for the treatment of RM-NENs. Furthermore, the prognosis of patients with RM-NENs is still uncertain. Several studies have reported favorable short-term results after the endoscopic resection of RM-NENs smaller than 10 mm [13, 14]. Due to the small number of cases, only a few epidemiological reports are available for reference, and the clinicopathological features are not clear. It is also difficult to conclude whether the presence of multiple tumors is associated with lymph node metastasis. In summary, there is no consensus on the treatment of RM-NENs.

This study summarized the clinicopathological characteristics of RM-NENs in patients at our center, reviewed literature reports, and preliminarily discussed the clinical diagnosis and treatment of RM-NENs.

## Methods

### Clinical data collection

This study retrospectively analyzed R-NEN patients diagnosed and treated at the Fourth Hospital of Hebei Medical University from February 2007 to May 2021. The exclusion criteria were as follows: 1) R-NENs combined with other types of colorectal cancer and 2) R-NENs combined with other malignant tumors. This project was approved by the Ethics Committee of the Fourth Hospital of Hebei Medical University (ID: 2021KS002).

### Literature search

A comprehensive search strategy was adopted to include as many relevant studies as possible. The PubMed and Chinese academic publication websites were searched for articles published from inception until May 2021. The search term combinations were Medical Subject Heading (MeSH) terms, text words, and variants of neuroendocrine tumors, neuroendocrine neoplasms, carcinoid, rectal, and multiple. The reference lists of all retrieved articles were searched manually for other possible studies. In the retrieved articles, the cases of RM-NENs were recorded, and duplicate cases were excluded.

The clinicopathological characteristics of RM-NENs were summarized and analyzed in combination with 14 studies on RM-NENs.

### Data analysis

Statistical analysis was performed using IBM SPSS Statistics V. 25.0.0 (IBM Corp, New York). Continuous variables are expressed as the mean ± standard deviation (SD) or median and interquartile range, and statistical analysis was performed using a t test. Other data are expressed as numbers and percentages and were analyzed by the chi-square test. Two-tailed *p* values were used for all statistical tests, and *P* values < 0.05 were considered statistically significant.

## Results

In total, 183 patients with R-NENs were diagnosed between February 2007 and May 2021, including 176 patients (96.2%) with RS-NEN and 7 patients (3.8%) with RM-NENs.

**Fig. 1:**
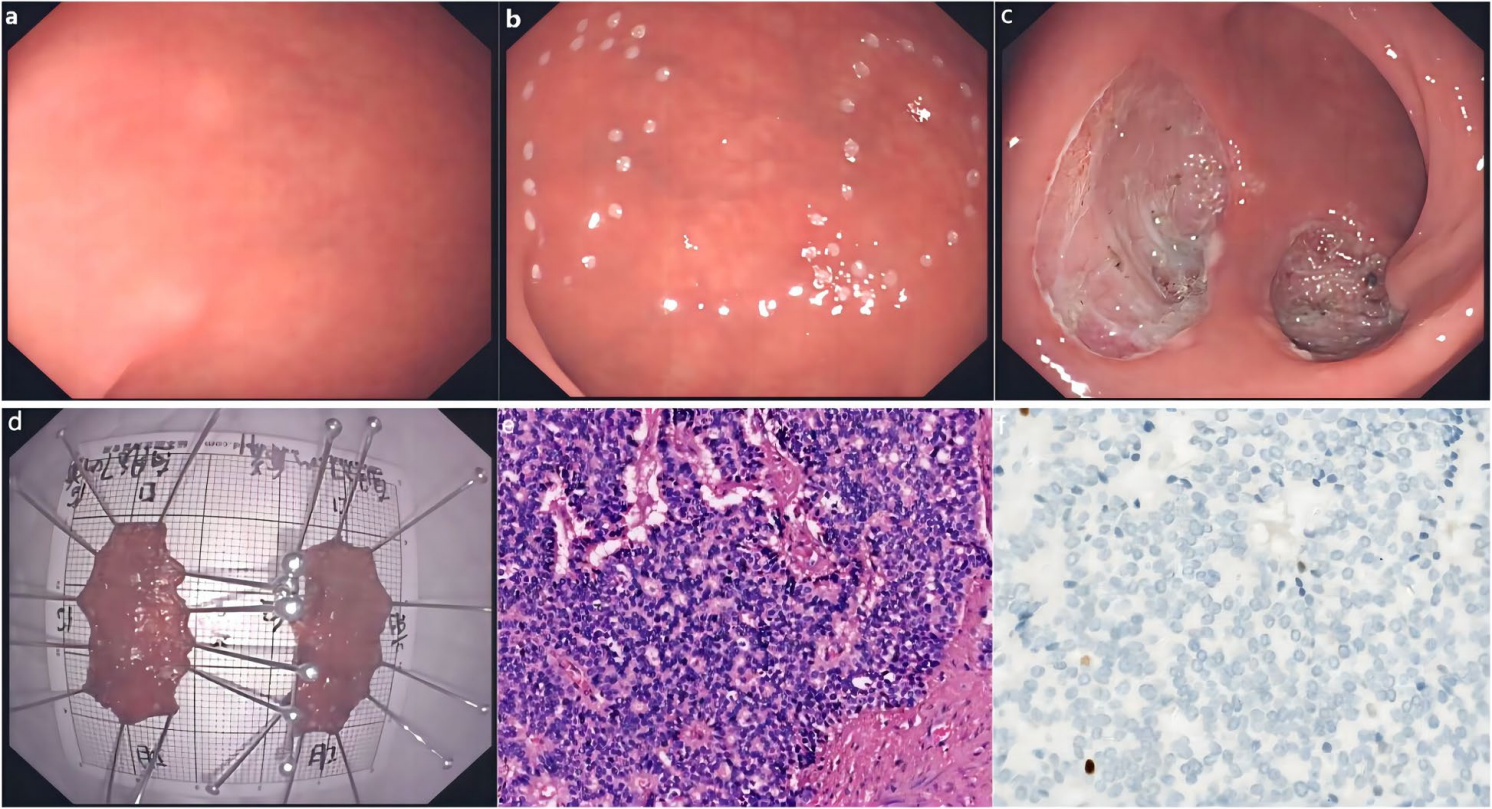
Endoscopic resection of RM-NENs: **a** Five tumors found in the rectum. **b** Marked preresection area. **c** Wound after tumor resection. **d** Fixed specimens. **e** HE staining (10×). **f** Ki-67 index of 1% (20×)

Among the patients with RM-NENs, there were 4 males (57.1%) and 3 females (42.9%), with a median age of 49 (46–69) years; there was a total of 17 tumors, including 2 tumors in 6 cases and 5 tumors in 1 case, all of which were less than 10 mm in size. All tumors invaded the submucosa. No lymph node metastasis or distant metastasis was found in the preoperative examination of any patient. All tumors were completely resected by endoscopic resection (Fig. 1). Thirteen tumors were grade G1, 2 were G2, and 2 was ungraded. The median follow-up was 92 (32 ~ 132) months. No tumor recurrence or metastasis was discovered on follow-up (follow-up ended in May 2021).

Combined with cases in the 14 reviewed studies, there were 47 cases of RM-NENs (Table 1). Among these patients (Table 2), 29 were males (61.7%), and 18 were females (38.3%), aged between 32 and 81 years. The tumor size was generally less than 10 mm, and the number of tumors ranged from 2 to 69. Thirty-four patients (72.3%) had fewer than 8 tumors, and 13 patients (27.7%) had more than 8 tumors. In 45 cases (95.7%), tumors invaded the submucosa, and in 2 cases (4.3%), tumors were confined to the mucosa. There were 29 (61.7%) patients with grade G1 tumors, 3 (6.4%) patients with grade G2 tumors, and 15 (31.9%) patients with ungraded tumors. There were 8 patients (17.0%) with lymph node metastasis, 38 patients (80.9%) without lymph node metastasis, and 1 patient (2.1%) with an unclear lymph node metastasis status. There were 44 patients (93.6%) without distant metastasis and 3 (6.4%) with an unclear distant metastasis status. In terms of the tumor treatment plan, follow-up was performed in 1 case (2.1%), the treatment was unspecified in 1 case (2.1%), and surgical excision (the specific method could not be determined) was performed in the remaining cases (95.8%).

**Table 1:** A bibliographic listing of RM-NEN reports

NO.	Cases	Gender	Age	Tumor Number	Size (mm)	Depth	Histology	Lymph node metastasis	Distant metastasis	Treatment
1	MICHAEL KANTER et al. [9] (1987, USA)	M	50	17	< 10	SM	carcinoid	Yes	No	Surgery
2	Sung Sil PARK et al. [10] (2018, Korea)	M	57	12	1–10	SM	G1	Yes	No	Surgery
3	Masashi Haraguchi et al. [15] (2007, Japan)	M	69	30	< 10	SM	carcinoid	No	No	Surgery
4	Kevin A. Ghassemi et al. [16] (2009, USA)	F	53	6	2–3	SM	carcinoid	No	No	Follow-up
5	Shunichi Sasou et al. [7] (2012, Japan)	M	51	69	< 8	SM	G1	Yes	No	Surgery
6	Shunichi Sasou et al. [7] (2012, Japan)	M	58	62	< 7	MP	G2	Yes	No	Surgery
7	Chan Seo Park et al. [17] (2014, Korea)	M	52	4	4	SM	G1	No	No	EMR-L
8	Chan Seo Park et al. [17] (2014, Korea)	M	32	3	5–7	SM	G1	No	No	EMR-L
9	Chan Seo Park et al. [17] (2014, Korea)	F	65	3	5–7	SM	NET	No	No	EMR
10	Chan Seo Park et al. [17] (2014, Korea)	M	62	2	5	SM	G1	No	No	EMR-L
11	Chan Seo Park et al. [17] (2014, Korea)	F	48	2	-	SM	G1	No	No	EMR-L
12	Jiao-Lin Zhou et al. [18] (2015, China)	M	47	3	5–8	SM	G1	No	No	TEM
13	Bai Hua et al. [19] (2016, China)	F	61	> 10	3–10	SM	G1	No	No	Surgery
14	Momoko Doi et al. [20] (2016, Japan)	M	61	42	1–6	SM	G1	No	No	Surgery
15	Momoko Doi et al. [20] (2016, Japan)	M	61	36	1–5	SM	G1	No	No	Surgery
16	Rui Xie et al. [21] (2018, China)	F	39	dense	3–25	SM	G1	Yes	No	Follow-up
17	M Kato et al. [22] (1986, Japan)	M	61	52	1–6	SM	-	-	-	-
18	Maruyama M et al. [8] (1988)	M	53	5	4–10	MP	-	No	-	Surgery
19	Okamoto Y et al. [23] (2004, Japan)	M	54	4	< 6	SM	-	No	-	EMR-L
20	Mine (2019, China)	M	46	2	5–6	SM	G1	No	No	ESD
21	Mine (2011, China)	F	49	2	4–8	SM	NET	No	No	EMR-C
22	Mine (2012, China)	F	50	2	6–10	SM	G1	No	No	EMR-C
23	Mine (2014, China)	F	48	2	4–10	SM	G1	No	No	EMR-C
24	Mine (2010, China)	F	48	2	6–8	SM	G2	No	No	EMR-C
25	Mine (2018, China)	M	69	2	4–8	SM	G1	No	No	ESD
26	Mine (2014, China)	M	53	5	3–8	SM	G1	No	No	ESD
27–47	Yusuke Nishikawa et al. [12] (2019, Japan)	M = 12 F = 9	59 (42–81)	2–27	< 10	SM	G1 = 13 G2 = 1	Yes = 3 No = 18	No	ER\Surgery

**Table 2:** Clinicopathological features of 47 patients with RM-NENs

Factors	Cases (*n* = 47)
**Gender**	
Male	29 (61.7%)
Female	18 (38.3%)
**Age**, years, range	32–81
**Tumor number**, range	2–69
< 8	34 (72.3%)
≥ 8	13 (27.7%)
**Tumor size**, mm	≤ 10
**Infiltration depth**	
SM	45 (95.7%)
Muscularis propria	2 (4.3%)
**Histology**	
Unknown	15 (31.9%)
G1	29 (61.7%)
G2	3 (6.4%)
**Lymph node metastasi**s	
Unknown	1 (2.1%)
No	38 (80.9%)
Yes	8 (17.0%)
**Distant metastasis**	
Unknown	3 (6.4%)
No	44 (93.6%)
Yes	0 (0)

In the analysis of 46 cases of known lymph node metastasis (Table 3), there was a significant difference in the lymph node metastasis rate between those with < 8 and ≥ 8 tumors (*p* = 0.002).

**Table 3:** Risk factors for lymph node metastasis in RM-NENs

Factors	Cases (*n* = 46)	Lymph node metastasis (*n* = 8)	*p*-value
**Gender**			0.453
Male	28	6 (21.4%)	
Female	18	2 (11.1%)	
**Tumor number**			0.002
< 8	34	2 (5.9%)	
≥ 8	12	6 (50%)	
**Infiltration depth**			0.321
SM	44	7 (15.9%)	
Muscularis propria	2	1(50%)	
**Histology**			0.099
Unknown	14	1 (7.1%)	
G1	29	5 (17.2%)	
G2	3	2 (66.7%)	

## Discussion

R-NENs are hindgut tumors. The pathogenesis of hindgut NENs has not been elucidated, especially at the molecular level. Studies suggest that endocrine cells in the crypt proliferate, infiltrate or migrate to the lamina propria, muscularis mucosae and submucosa and may develop into carcinoid cells, which may be multipotent [7, 8]. Pathological examination of R-NENs has shown that they are not multipotent. In addition, patients with rectal neuroendocrine tumors usually only have one, and only 2%-5.7% of patients have multiple tumors [8, 12]. In our study, the incidence of RM-NENs among R-NEN patients was 3.8%, which is consistent with literature reports. Previous studies have suggested that the MEN1 gene and PI3-K/AKT, Raf/MEK/ERK, Notch, GSK-3β and other signaling pathways may be involved in the occurrence and metastasis of multiple rectal tumors [24]. It has also been reported that RM-NENs may be associated with inflammatory bowel disease [25]. Hiripi et al. [26] reported a significantly increased risk of NENs in individuals with a parental history of such tumors. Momoko Do et al. [20] reported multiple neuroendocrine tumors in identical twins at the same location (rectum), suggesting that the occurrence of tumors is related to genetic background.

Clinical evidence [27, 28] has shown that when the size of an RS-NEN is less than 10 mm, lymph node metastasis is rarely observed, and the metastasis rate is less than 10%. However, when the R-NEN diameter is larger than 20 mm, the lymph node metastasis rate can be as high as 60%-80%, and the distant metastasis rate can be up to 40%. Surgical excision is considered the most appropriate treatment. Standard surgical methods [14, 29–37] include endoscopic submucosal dissection (ESD), endoscopic mucosal resection (EMR), transanal resection of the mass, and surgical resection. The choice of surgical method depends on the tumor size, depth of invasion, regional lymph nodes, distant metastasis, and malignancy. Meanwhile, endoscopic ultrasonography (EUS) [38–40] can be used to determine the size, depth of invasion and metastasis status of adjacent lymph nodes and detect whether a submucosal mass is separate from the muscularis propria, which is crucial in deciding the treatment plan and evaluating the stability of endoscopic resection. However, since EUS cannot be used to determine the distant lymph node or liver metastasis status, abdominal CT, MRI, and PET-CT examinations are required to evaluate distant metastasis.

Due to the rarity of RM-NENs compared to RS-NEN, there are no standard treatment guidelines. In addition, the long-term prognosis of RM-NENs patients after endoscopic resection remains uncertain. However, treatment can be performed based on the size and depth of infiltration of each R-NEN. Multiple tumors were successfully resected by endoscopy in 7 patients, with an excellent short-term prognosis and no local recurrence. However, due to the slow growth of R-NENs, it is difficult to evaluate the long-term efficacy of or prognosis after endoscopic resection [17].

In small intestinal carcinoid tumors, polycentricity is a poor prognostic factor [41, 42]. However, its prognostic effect in R-NENs is unclear. It has been reported that the incidence of lymph node metastasis is very high in patients with multiple tumors, regardless of tumor size and pathological grade [20]. Yusuke Nishikawa et al. [12] showed in a single-center retrospective analysis that the overall lymph node metastasis rate of RM-NENs was 14.3% and increased to 33.3% when the number of tumors was ≥ 8. In our study, lymph node metastasis occurred in 17% of patients, but no distant metastasis was observed. This is also an extremely rare situation; due to the extensive proliferation of neuroendocrine cells in the rectum, the number of tumor lesions could not be estimated because they could not be seen by the naked eye. Therefore, surgical resection was required [7, 8]. However, how can the number of tumors in RM-NENs patients be determined? There is no size criterion to distinguish neuroendocrine tumors from endocrine cell micronests (ECMs). According to the judgment of senior pathology teachers, 7 cases in our center did not have ECMs. Based on the description and picture information in the literature reviewed, we conclude that there may be 10 cases of suspected ECMs, which needs to be carefully judged. In upper gastrointestinal neuroendocrine tumors, lesions larger than 0.5 mm meeting the immunohistochemical criteria can be diagnosed as tumors. In gastric neuroendocrine tumors, tumor cells mainly develop from gastric mucosa intestinal chromaffin cells [43]. Gastrinemia induces the proliferation of intestinal chromaffin cells, leading to type I gastric neuroendocrine tumors, which are prone to be accompanied by ECMs [44–48]. Because cases of R-NENs with ECMs are so rare, it is not clear whether treatment for these ECMs is needed. Maruyama et al. [8, 48] described the possible origin of ECMs for R-NENs; however, it is not clear whether glandular endocrine cells are derived from the neuroectoderm along the nerve fibers or endoderm stem cells. It has also been speculated that ECMs can be regarded as the initial or intermediate stage of carcinoid development [8]. In addition, it has previously been reported that ECMs might be a marker of the presence of multiple carcinoid and lymph node metastases [7, 10]. In contrast, Wong et al. [25] argued that ECMs do not appear to develop into neuroendocrine tumors and may not require further clinical examination or invasive procedures, including endoscopic resection, during surveillance and noted that inflammatory bowel disease is prone to cause neuroendocrine cell proliferation. Sho Suzuki et al. [44] reported that multiple ECMs existed around an RS-NEN lesion. No lymph node metastasis or distant metastasis was found on CT examination 6 years after endoscopic resection. Because case reports of multiple ECMs are very rare, many aspects of ECMs remain unclear to date, and the significance of malignancy is unclear; thus, further studies are needed to confirm the role of ECMs.

It is unclear whether the biology and behavior of RM-NENs are consistent with those of the largest lesion, as a cumulative tumor burden, or whether the number of tumors affects prognosis. The treatment plan should be individualized in each case, and careful follow-up is needed. Clinicians should aim to improve the understanding of this disease, which requires early detection and treatment.

Of course, there are some limitations to our study. First, this was a single-center retrospective study, and there may have been bias in the selection of cases. Second, the sample size was relatively small. Therefore, a large prospective randomized controlled trial is needed to investigate RM-NENs.

In conclusion, RM-NENs accounted for 3.8% of all R-NENs in this study. The number of tumors varied to some extent, most tumors were no more than 10 mm in size, and there were more grade G1 tumors. For RM-NENs, the lymph node metastasis rate was higher when the number of tumors was ≥ 8. The influence of the number of tumors on lymph node metastasis should be considered in the selection of treatment.

## Data Availability

The datasets used and analyzed during the current study are available from the corresponding author upon reasonable request without breaching participant confidentiality.

## Abbreviations

R-NEN: Rectal neuroendocrine neoplasm
RM-NENs: Rectal multiple neuroendocrine neoplasms
RS-NEN: Rectal single neuroendocrine neoplasm
EMR: Endoscopic mucosal resection
ESD: Endoscopic submucosal dissection
EUS: Endoscopic ultrasonography
ECMs: Endocrine cell micronests
